# *STAT1* gain-of-function variant in a previously healthy patient with disseminated *Mycobacterium genavense* infection

**DOI:** 10.70962/jhi.20250139

**Published:** 2026-08-25

**Authors:** Kavitha Thiagarajan, Huan Vinh Dong, Caroline Y. Kuo, Timothy J. Thauland, Sheeja Pullarkat, Manish J. Butte, Maria I. Garcia-Lloret, Karin Nielsen-Saines

**Affiliations:** 1 Division of Allergy, Immunology & Rheumatology, Department of Pediatrics, University of California, Los Angeles, Los Angeles, CA, USA; 2 Pediatric Infectious Diseases, David Geffen School of Medicine, University of California, Los Angeles, Los Angeles, CA, USA; 3 Department of Pathology, University of California, Los Angeles, Los Angeles, CA, USA; 4 Division of Pulmonary, Critical Care, Allergy, and Sleep Medicine, Department of Internal Medicine, University of California, San Francisco, San Francisco, CA, USA

## Abstract

Signal transducer and activator of transcription 1 (STAT1) defects present with diverse clinical phenotypes. We describe an unusual genotype/phenotype: disseminated mycobacterial infection without chronic mucocutaneous candidiasis in the context of a pathogenic STAT1 gain-of-function variant (p.Lys388Glu). To our knowledge, this is the first report of such a case.

## Teaser

STAT1 defects present with diverse clinical phenotypes. Thiagarajan et al. describe an unusual genotype/phenotype: disseminated mycobacterial infection without CMC in the context of a pathogenic STAT1 GOF variant (p.Lys388Glu).

## Introduction

Signal transducer and activator of transcription 1 (STAT1) is a transcription factor encoded by the *STAT1* gene. STAT1 is essential for T helper (Th) 1–mediated immunity and defense against viral, mycobacterial, and other intracellular organisms by mediating the cellular response to IFNs, including IFN-γ. The IFN-γ and IL-12 pathway is one essential component for host defense against mycobacterial infections (1).

To date, there have been more than 400 published cases of STAT1 gain-of-function (GOF) with >100 different inborn variants (2). Approximately 93% of patients developed chronic mucocutaneous candidiasis (CMC) in the first decade of life (2). In contrast, disseminated mycobacterial infections were extremely rare (2). We describe the clinical, genetic, and immunological features of a previously healthy young female without CMC who presented with disseminated *Mycobacterium genavense* infection in the setting of a pathogenic, GOF *STAT1* variant. We also highlight the utilization of the Karius assay in microbial diagnosis and monitoring.

## Results

### Case description

The patient consented to submission of the case report.

A 19-year-old female presented with 3 months of abdominal pain, weight loss, fevers, and night sweats. She had no history of serious illnesses or hospitalizations. She denied any oral, esophageal, or vaginal candidiasis. She primarily resided in California but lived in Florida for 2 years. She was of Korean and Japanese descent. She had no siblings; her parents were healthy, and there was no history of consanguinity. She had not received any vaccinations in accordance with her religious beliefs.

Physical exam on initial presentation demonstrated cachexia, marked hepatosplenomegaly, and significant abdominal tenderness. Her oropharynx was normal without evidence of thrush.

Initial labs were notable for a white blood cell count of 12 k/μl with CD4 T cell lymphopenia (CD4^+^ 140/μl, normal 441–2,156/μl). B and natural killer (NK) cells were normal. Hemoglobin (Hgb) was 9.7 g/dl (normal 12–16 g/dl), and C-reactive protein was 168 mg/L (normal <10 mg/L). HIV antigen and antibody testing were negative. Liver enzymes were within normal limits.

Infectious workup was largely unrevealing, with cultures of blood, sputum, and bone marrow showing no growth. A biopsy of an enlarged lymph node was obtained and cultured without growth of an organism after over 30 weeks of incubation. Her stool showed staining for acid-fast bacilli (AFB). The pathogen was only identified as *M. genavense* by metagenomic testing from cell-free DNA (cfDNA). Malignancy was ruled out by bone marrow and lymph node biopsies.

**Figure 1.**
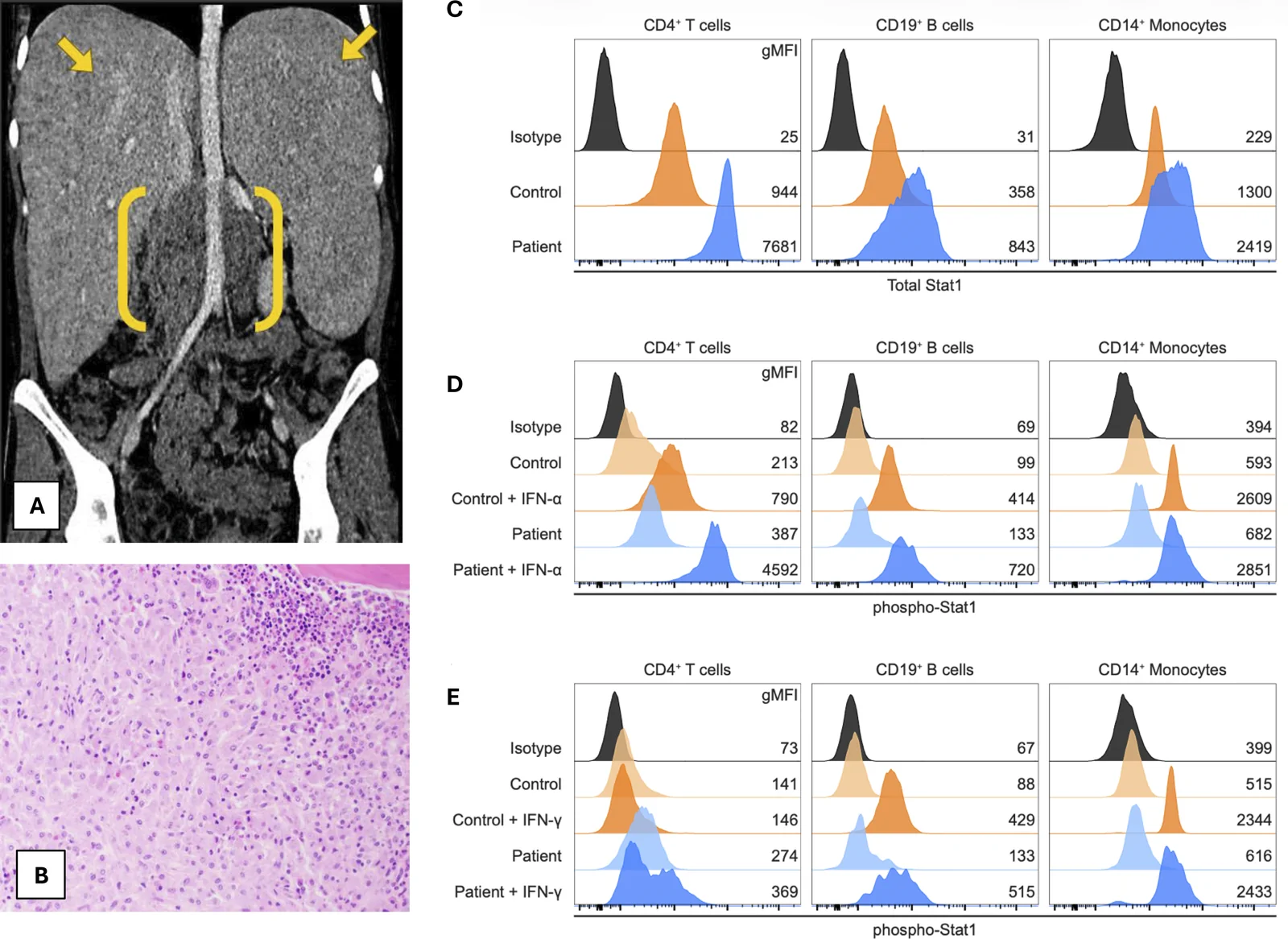
**CT abdomen/pelvis showing hepatosplenomegaly and lymphadenopathy, histology from the bone marrow biopsy showing sheets of histiocytes and decreased hematopoiesis, and elevated total and phospho-STAT1 levels in our patient’s T and B lymphocytes and monocytes, especially the T lymphocytes.** **(A)** CT of abdomen demonstrated hepatomegaly (25 cm in the craniocaudal imension) (left arrow), splenomegaly (22 cm craniocaudal dimension) (right arrow), and massive lymphadenopathy around the aorta (with a conglomerate measuring 71 × 94 mm in upper central mesentery encasing the abdominal aorta) (brackets). **(B)** Bone marrow biopsy H&E section (400× magnification) revealed sheets of histiocytes characterized by abundant eosinophilic cytoplasm and round to oval nuclei. There is a markedly decreased presence of normal hematopoiesis. **(C)** Flow cytometry showed patient cells had higher levels of total STAT1 than controls in all cells tested, including CD4^+^ T cells, CD19^+^ B cells, and CD14^+^ monocytes (representative of three experiments from PBMC collected at different time points). **(D)** IFN-α stimulation found that patient cells had higher levels of phospho-Stat1 than controls. This was especially pronounced in CD4^+^ T cells. Results representative of three experiments from PBMC collected at different time points. **(E)** IFN-γ stimulation found that patient cells had higher levels of phospho-Stat1 than controls. This was especially pronounced in CD4^+^ T cells. Results representative of three experiments from PBMC collected at different time points.

Abdominal computed tomography (CT) confirmed hepatosplenomegaly and multi-station lymphadenopathy, most significant within the mesenteric plexus (Fig. 1 A).

### Treatment course

The patient was started on ethambutol, rifampin, and azithromycin but could not tolerate therapy due to severe nausea and abdominal pain. She was lost to follow-up but returned to care after 4 months with 70-pound weight loss due to persistent abdominal pain and difficulty with oral intake. She required inpatient admission for profound electrolyte derangements and pancytopenia.

Laboratory results at this time were significant for Hgb as low as 5.0 g/dl (normal 12–16 g/dl) and platelets as low as 9,000/μl (normal 143,000–398,000/μl), requiring numerous red blood cell and platelet transfusions. There was no evidence of hemolysis or autoimmune cytopenias. During this time, her lymphopenia worsened significantly, with CD4^+^ T cells as low as 45 cells/μl, with B and NK lymphopenia as well. Bone marrow biopsy showed decreased hematopoiesis and abundant CD163^+^ macrophages. AFB stain demonstrated innumerable AFB within these macrophages.

Further immune evaluation showed profound T cell activation with type 1 skewing (serum soluble IL-2 receptor [CD25] 4,000 pg/ml [normal 175–858 pg/ml] and serum CXCL9 15,400 pg/ml [normal <179 pg/ml], indicative of excessive IFN-γ signaling). Her humoral immunity was intact with normal serum immunoglobulin levels and detectable antibody titers to *Streptococcus pneumoniae* and tetanus, despite lack of documented childhood vaccinations. She also had a normal neutrophil count with normal granulocyte oxidative burst.

Repeat cfDNA testing revealed a drastic increase in *M. genavense* burden, rising from 502 molecules of microbial cell-free DNA per microliter (MPM) to 178,993 MPM. Given the inability to obtain antimicrobial susceptibilities, the patient was empirically treated with amikacin, azithromycin, ethambutol, and rifampin. Trimethoprim-sulfamethoxazole prophylaxis was initiated in the setting of profound CD4 lymphopenia.

Trio exome sequencing revealed a heterozygous de novo p.Lys388Glu (NM_007315.3:c.1162A>G) pathogenic variant in *STAT1*. Evaluation of IFN-stimulated STAT1-phosphorylation showed a GOF profile, particularly in T lymphocytes; this pattern was present but less prominent in B lymphocytes and monocytes (Fig. 1, D and E). Total STAT1 expression was also elevated across immune cell populations, further supporting the diagnosis of STAT1 GOF (Fig. 1 C).

Given the STAT1 GOF profile, JAK 1/2 inhibition with low-dose baricitinib was started 1 year following initiation of antimycobacterial therapy, once she was more clinically stable. After a month of baricitinib, the hepatosplenomegaly on physical exam dramatically improved, the anemia resolved, and serum soluble IL-2 receptor normalized. Serum CXCL9 remained elevated but overall improved (8,020 pg/ml). CD4^+^ T cell count improved to 114/uL. Notably, the cfDNA assay no longer detected circulating *M. genavense* for the first time.

## Discussion

Inborn variants in *STAT1* are associated with a wide spectrum of clinical phenotypes. GOF variants confer susceptibility to CMC, as well as autoimmunity, vascular aneurysms, and malignancy. These variants are typically inherited in an autosomal dominant manner but may also arise de novo (1, 2). A recent meta-analysis demonstrated that of 442 patients with STAT1 GOF, 93% developed CMC before the age of 10, while <10% had mycobacterial disease, with only one reported case of disseminated *M. genavense* (2). Additionally, approximately one-third of the patients had CD4 T cell and NK lymphopenia (2, 3). In contrast, loss-of-function (LOF) variants can be monoallelic or biallelic and are associated with susceptibility to mycobacterial, fungal, and viral infections (2). Our patient’s presentation was more consistent with LOF given the absence of CMC, autoimmunity, and aneurysms on imaging. The presence of CD4 lymphopenia likely predisposed her to infection with *M*. *genavense*.

*M*. *genavense* is a fastidious, slow-growing, nontuberculous mycobacterium (NTM) present in tap water, animals, and the healthy human gastrointestinal tract (4). Infections with *M. genavense* are primarily described in immunocompromised hosts, historically in patients with uncontrolled HIV (4). Clinical presentation is similar to other NTM infections, with fever, lymphadenopathy, hepatosplenomegaly, and pancytopenia due to bone marrow invasion.

The normal immune response to mycobacteria relies on the activation of macrophages and subsequent engulfment of infected cells, primarily mediated through the IL-12/IFN-γ signaling pathway.

The p.Lys388Glu variant affects the DNA-binding domain of STAT1 and has been previously characterized as GOF in one patient with CMC (5). Among the STAT1 GOF patients in the meta-analysis by Zhang et al., almost all patients possessed missense variants in the coiled-coil and DNA-binding domains, although the p.Lys388Glu was not reported (2). At the cellular level, the GOF profile in our patient was demonstrated primarily in T lymphocytes but was still present in B lymphocytes and monocytes. This hyperphosphorylation of STAT1 results in impaired development of Th17 cells and poor candidal killing, which is why the majority of STAT1 GOF patients present with CMC.

It remains unclear why overactivation of STAT1 would also result in impaired mycobacterial killing. One possibility is that STAT1 overactivity results in upregulation of suppressor of cytokine signaling 1 (SOCS1), which could dampen responses to a surge of IFN-γ that might be needed to control mycobacterial infection.

Regarding immunomodulatory treatment options, hematopoietic stem cell transplantation has been attempted, but prior to the use of JAK inhibitors, it was complicated by high mortality and high rates of secondary graft loss (1). Our patient would also not be a candidate for transplant given the inability to eradicate the mycobacterial infection, risking further dissemination with myeloablation. Oral JAK inhibition is a safer option but carries its own risks. JAK inhibitors have been effective for reducing CMC and autoimmune manifestations of STAT1 GOF, including autoimmune cytopenia and thyroid disease (1, 2). The effect of JAK inhibition in disseminated infections needs further study.

This case also illustrates the use of cfDNA to trend quantitative levels of circulating microbial DNA fragments in the blood. In our patient, quantification of *M. genavense* by the Karius assay correlated with clinical improvement. However, the clinical utility of trending these values across different types of infections remains unclear, as this approach has not been systematically studied.

From a pathology perspective, the bone marrow biopsy was significant for innumerable intracellular AFB and sheets of histiocytes with a lack of hematopoiesis. While it is likely that the *STAT1* variant and consequent CD4 T cell lymphopenia predisposed her to the infection, we suspect that the mycobacteria’s ability to invade and proliferate in the bone marrow worsened her pancytopenia.

Fortunately, our patient has shown continued improvement with antimicrobial therapy and low-dose JAK 1/2 inhibition (baricitinib 2 mg daily). Continued monitoring of her lymphopenia, inflammatory markers, total/phosphorylated STAT1 levels, splenomegaly, and mycobacterial burden will determine ongoing clinical response to JAK inhibition. Ultimately, one should expect the unexpected in patients with STAT1 GOF.

## Data Availability

The data underlying Fig. 1 are available in the published article. The ClinVar accession number for this case is SCV007613769.1.
